# *LiaS* gene from two-component system is essential for caries pathogenicity in dual-species biofilms of *Streptococcus mutans* and *Candida albicans*

**DOI:** 10.3389/fmicb.2025.1612841

**Published:** 2025-07-31

**Authors:** Yanfan Cao, Yu Zeng, Yuze Yang, Guowu Gan, Shuai Chen, Xiaojing Huang

**Affiliations:** Fujian Key Laboratory of Oral Diseases and Fujian Provincial Engineering Research Center of Oral Biomaterial and Clinical Research Center for Oral Tissue Deficiency Diseases of Fujian Province, School and Hospital of Stomatology, Fujian Medical University, Fuzhou, China

**Keywords:** *Streptococcus mutans*, *Candida albicans*, *liaS*, two-component system, biofilm, extracellular polysaccharide

## Abstract

**Introduction:**

This study elucidated the critical role of the *liaS* gene in the *Streptococcus mutans* (*S. mutans*) two-component signal (TCS) transduction system during cross-kingdom interactions with *Candida albicans* (*C. albicans*). This gene governs the cariogenic potential of dual-species biofilms.

**Methods:**

Gene expression analysis of cocultured samples was performed. The survival rate during H_2_O_2_ treatment in single and dual-species was assessed. Bacterial adhesion, extracellular polysaccharide (EPS) synthesis, acidic metabolite accumulation, and early adhesion of *C. albicans* in dual-species biofilms were evaluated.

**Results:**

Compared with the wild-type (*WT*) and complemented strains (liaS^–^-comp), liaS- exhibited impaired acid tolerance due to downregulated comDE expression. The *liaS* knockout mutant strain also presented reduced *vicRK* expression, leading to diminished glucosyltransferase (Gtf)-mediated bacterial adhesion, EPS synthesis, and acidic metabolite accumulation. Although *C. albicans* alleviated oxidative stress by secreting superoxide dismutase, liaS- markedly compromised the intracellular reactive oxygen species (ROS) scavenging capacity and reduced the survival rate during H_2_O_2_ treatment in single and dual-species. Furthermore, liaS- inactivation suppressed the early adhesion of *C. albicans* in dual-species biofilms by reducing the synthesis of cyclic adenosine monophosphate (cAMP).

**Conclusion:**

This work provides the first evidence that *liaS* orchestrates a multidimensional phenotypic regulatory network that coordinately modulates biofilm architecture and metabolic activity. This activity ultimately attenuates cariogenicity *in vivo*, thus highlighting liaS as a pivotal virulence determinant in cross-kingdom infections and emphasizing its potential as a therapeutic target against dental caries.

## Introduction

Dental caries is one of the most prevalent diseases worldwide and is characterized by the chronic progressive destruction of hydroxyapatite mediated by cariogenic microorganisms and complex biofilms (Sabharwal et al., 2021). Several reports have highlighted a correlation between dental caries or apical periodontitis and systemic diseases (Sabharwal et al., 2021). Therefore, the prevention and management of dental caries are highly important for improving global health.

The microbiological etiology of dental caries involves synergistic interactions among multiple bacterial species, with *S. mutans* recognized as the key cariogenic pathogen driving caries development (Homayouni Rad et al., 2023). The bacterium adheres to tooth surfaces through sucrose-dependent adhesion mechanisms, thus forming structured, acidogenic biofilms. Furthermore, through glycolysis, *S. mutans* metabolizes dietary carbohydrates to produce lactic acid, which progressively acidifies the biofilm microenvironment to a pH below 4.5. This acidic milieu induces dissociation of hydroxyapatite crystals and triggers the progressive loss of calcium and phosphate ions, thereby serving as the primary driver of enamel demineralization.

The cariogenic potential of *S. mutans* is primarily based on Gtf, which hydrolyzes sucrose into glucose and fructose, followed by glucose polymerization via glycosidic bonds to drive the biosynthesis of EPS (Lahiri et al., 2021). The three-dimensional (3D) EPS network not only functions as a biofilm scaffold to promote microbial coaggregation but also reduces antimicrobial penetration through steric hindrance effects (Flemming et al., 2023). Upon interaction with extracellular DNA (eDNA), EPS enhances the mechanical stability of biofilm and facilitates horizontal gene transfer, thereby accelerating the emergence of antibiotic resistance mutations (Zhang et al., 2021). Genetically, Gtfs are encoded by the *gtfB*, *gtfC*, and *gtfD* genes, each of which play distinct functional roles: GtfB catalyzes α-1,3-glycosidic bonds to produce insoluble glucans for structural support; GtfC synthesizes α-1,6/α-1,3-mixed linkage glucans that mediate microbial coaggregation; and GtfD generates soluble glucans involved in glycogen storage and pH homeostasis (Paes Leme et al., 2006). Targeted inhibition of Gtfs to reduce EPS synthesis has emerged as a promising strategy for developing anti-caries therapies that preserve the oral microbial equilibrium.

*C. albicans*, a dimorphic commensal-pathogenic fungus in the oral cavity, regulates its virulence through yeast-to-hyphae morphological transitions, while its pathogenicity is modulated by interspecies interactions (Man et al., 2025). Studies have demonstrated that *Streptococcus sanguinis* can increase *C. albicans* biofilm formation via metabolic cross-feeding mechanisms, while *Streptococcus gordonii* secretes competence-stimulating peptide (CSP) to activate the fungal cAMP-protein kinase A signaling pathway, thereby upregulating hyphae-specific gene expression (Du et al., 2021; Man et al., 2025). Within cariogenic microbiomes, *C. albicans* and *S. mutans* can establish synergistic consortia. Fungal cell wall α-mannans can directly bind to *S. mutans*-derived GtfB, thus enabling bacterial colonization on hyphal surfaces. Sucrose hydrolysis releases fructose, which is assimilated by *C. albicans* to activate the MAPK pathway through Hxk1 kinase-mediated signaling (Pierce et al., 2017). The cross-kingdom consortium secretes pyruvate, thus promoting hydroxyapatite dissolution through calcium chelation. Simultaneously, fungal phospholipase B2 disrupts the hydrophobic pellicle, thereby exposing enamel crystals to acid-driven demineralization (Nikawa et al., 2003). These interspecies interactions highlight the potential role of cross-kingdom symbiosis in driving ecological shifts toward cariogenic states.

The two-component signal (TCS) transduction system is a pivotal regulatory mechanism through which *S. mutans* modulates its cariogenic phenotype. Studies have demonstrated that the TCS coordinates biofilm formation, EPS biosynthesis, and quorum sensing (QS) to increase environmental adaptability and cariogenicity. Notably, the TCS network also operates through non-canonical pathways. Recent studies have revealed the distinctive role of the *liaSR* system in environmental stress responses. The LiaSR two-component regulatory system is a widespread signaling pathway in gram-positive bacteria, including *S. mutans*. This system comprises two core components: a membrane-bound histidine kinase receptor (LiaS) and a corresponding response regulator (LiaR). LiaS functions as a sensor for environmental cues, initiating a signaling cascade via autophosphorylation and subsequently transferring the phosphoryl group to LiaR upon detection of stress signals. Activated LiaR specifically binds to promoter regions of the acid-resistant transporter gene SMU.753 and the antioxidant enzyme-encoding gene SMU.2084 (Suntharalingam et al., 2009; Tremblay et al., 2009; Qureshi et al., 2014). Studies have revealed the role of the LiaSR system in multiple physiological processes in *S. mutans*, including cell division, acid tolerance, biofilm formation, and antibiotic resistance, thus highlighting the system’s potential role as a regulatory hub in coordinating stress responses and virulence traits in a single oral pathogen. However, recent research has focused predominantly on monoculture regulatory mechanisms, leaving the functional reprogramming of *liaSR* in polymicrobial interactions and complex biofilm microenvironments largely unexplored.

Our group previously isolated a clinical strain (*S. mutans* 593) from caries-active individuals (DMFT ≥ 6). *In vitro* cariogenicity assessments revealed that, compared with *S. mutans* UA159, *S. mutans* 593 presented significantly greater cariogenic functional expression. Comparative analyses of *liaS* and *liaR* knockout mutant strains revealed that, compared with *WT*-*S. m* 593 and liaR^–^, liaS^–^ presented significantly reduced acid tolerance (Huang et al., 2024). However, whether *liaS* influences cariogenic potential through polymicrobial interactions remains unexplored. This study presents the first investigation of *liaS*-mediated regulatory effects on *S. mutans* 593-*C. albicans* biofilms, and further elucidates the cariogenic effects *in vivo*.

## Materials and methods

### Bacterial strains and biofilm formation conditions

The microorganisms used in this study included *C. albicans* SC5314, *S. mutans* 593, and liaS^–^, which were obtained from the School of Stomatology, Fujian Medical University (Fuzhou, China). The liaS^–^ strain and its complemented strain (liaS^–^-comp) were derived from *S. mutans* 593, using the construction methods and cultivation conditions described in previous studies (He et al., 2020; Alomeir et al., 2023). The dual-species coculture medium consisted of TYE broth containing 25 g/L tryptone (Oxoid, UK), 15 g/L yeast extract (Oxoid, UK), and 1% (w/v) sucrose (He et al., 2020). All the cultures were maintained at 37°C under 5% CO_2_.

For biofilm formation, pretreated *C. albicans* (2 × 10^4^ CFU/ml) and *S. mutans* (2 × 10^6^ CFU/ml, including liaS^–^ and liaS^–^-comp) were mixed at a 1:1 (v/v) ratio in TYE medium. The monospecies controls were grown under an equivalent system with 50% diluted medium (Rocha et al., 2018). The inoculated microplates were incubated for 24 h (37°C, 5% CO_2_), and the biofilm coverage was dynamically monitored using a cell analysis system (Zen CELL Owl, Leica Microsystems).

### Biofilm formation and biomass assay

A 24 h biofilm was constructed in a 96-well plate, and the crystal violet (CV) staining method was used to assess biofilm formation. After the supernatant was removed and the plates were washed with PBS, the biofilms were fixed in methanol for 15 min and stained with 0.1% (w/v) CV for 10 min. Following washing and drying, the biofilms were photographed using an optical microscope (ZEISS Stemi 508). The biofilms were then dissolved in 33.33% (v/v) acetic acid, and 100 μl of the solution was transferred to a new well plate for quantification on a microplate reader at OD575 nm (SpectraMax iD3). Biofilm samples were collected using sterile surgical blades, dissolved in PBS, and sonicated for 5 min to disperse the aggregates. Single-species and cocultured biofilm gradients were diluted and plated on sabouraud dextrose agar (SDA, Solarbio, China) and brain heart infusion (BHI, OXIOD, UK) agar to quantify *C. albicans* and *S. mutans*, respectively. After 48 h of incubation at 37°C, the biomass was evaluated. Each test was performed in triplicate, three times independently, and the average log_10_ CFU was used to determine the fungal and bacterial biomass (Alomeir et al., 2023).

### Acid tolerance response and production

To evaluate the impact of the *liaS* gene on the acid tolerance response (ART) of *S. mutans*, modified protocols from previous studies were employed (Turner et al., 2020; Du et al., 2023). In brief, the overnight-cultured microorganisms of *C. albicans* and *S. mutans* were mixed at the aforementioned concentrations and ratios to prepare single-species and dual-species planktonic cultures. The test samples were exposed to acidic TYE culture medium (initial pH 5.5) adjusted with 0.1 M HCl for 24 h, whereas the control samples were maintained in a neutral environment (pH 7.0). The single-species and cocultured mixtures were inoculated into sterile centrifuge tubes and incubated at 37°C in a shaking incubator (150 rpm) to maintain a planktonic cell suspension. The pH of the culture medium was not artificially adjusted during the experiment to allow for natural acidification via microbial metabolism. Biomass quantification was performed by serial dilution plating on SDA and BHI agar. After 48 h of incubation at 37°C, the viable colonies (30–300 CFU/plate) were counted (Jafri et al., 2020; Guo et al., 2023). The results from three replicates are expressed as the log_10_ CFU/ml.

The acid production capacity was assessed using a pH drop assay (Li et al., 2022; Lin et al., 2022a). The pH of the biofilm supernatants was measured using an Orion Dual Star pH/ISE benchtop meter (Thermo Scientific), which reflects the acid production from the glycolytic metabolism of carbohydrates by single and dual-species.

### Biofilm structural characterization

A multiparametric analysis of biofilms was conducted using confocal laser scanning microscopy (CLSM, Leica DM IRE2, Germany). The biofilms were developed on sterile glass slides (*r* = 0.6 cm) under anaerobic conditions. During initial cultivation, Alexa Fluor^®^ 647 (1.0 μM, ex/em: 650/668 nm; Molecular ProbesTM, Invitrogen) was added to label the EPS. After 24 h of incubation at 37°C, the planktonic cells were removed using PBS. Viable cells in biofilms were subsequently stained with SYTO^®^ 9 (2.5 μM, ex/em: 480/500 nm; Molecular ProbesTM, Invitrogen) for 15 min. Z-axis imaging was performed using a 40× oil immersion objective, with random field selection ensured through systematic sampling protocols. Biofilm thickness quantification was achieved using IMARIS V9.0 software (Bitplane AG, Zurich, Switzerland) that had built-in algorithms (Krzyściak et al., 2017; Liu et al., 2018; Sun et al., 2024).

### Oxidative stress and ROS assay

The antioxidant stress capacity mediated by *liaS* in single and dual-species biofilms was assessed using time-H_2_O_2_ killing curves (Yadav et al., 2014; Graziano et al., 2015). Mature 24 h biofilms were washed twice with PBS and treated with 2× minimum inhibitory concentration (MIC). The MIC values are provided in Supplementary Table 2. After incubation at 37°C for 0, 15, 30, 45, or 60 min, the reactions were terminated using PBS containing 1.0 mg/ml catalase (Graziano et al., 2015). The biofilms were harvested for CFU counting as previously described. The survival rates of *C. albicans* and *S. mutans* were calculated to compare oxidative stress resistance across biofilm systems.

Intracellular ROS levels were quantified using 2′,7′-dichlorodihydrofluorescein diacetate (DCFH-DA). The biofilms were loaded with 10 μM DCFH-DA probe and incubated at 37°C in the dark for 60 min. Cellular esterases convert the non-fluorescent probe to DCFH, which subsequently reacts with ROS to generate fluorescent 2′,7′-dichlorofluorescein (DCF; ex/em: 485/535 nm). The fluorescence intensity (FI) was measured using a microplate reader (SpectraMax i3x, Molecular Devices). Data were normalized to biofilm biomass (CFU counts) and expressed as FI/10^3^ cells to indicate ROS production per unit biomass.

### RNA extraction and RT-PCR

Ribonucleic acid (RNA) was extracted from *S. mutans* and *C. albicans* in dual-species biofilms using TRIzolTM reagent (Invitrogen, USA) according to the manufacturer’s protocol. Next, 1.0 μg of RNA was reverse-transcribed with a PrimeScriptTM RT reagent kit (Takara Biotechnology, Japan). The primers (Sangon Biotech, Shanghai, China) used in this study are shown in Supplementary Table 1. RT-PCR was performed on cDNA using a StepOnePlusTM Real-Time PCR System (Applied Biosystems, USA) with TB GreenTM Premix Ex TaqTM II (Takara Biotechnology, Japan). The *16S rRNA* and *18S rRNA* were used as housekeeping genes, and gene expression was quantified via the 2^–ΔΔCt^ method (Nailis et al., 2006).

### cAMP content in biofilms and early fungal adhesion

Cyclical liquid nitrogen freeze-thaw cycles were performed to increase the cAMP extraction efficiency. Following lyophilization and dry weight measurement, the samples were reconstituted in 200 μl cold PBS and subjected to 10 min of ultrasonication for cellular lysis. After centrifugation (5000 × *g*, 10 min, 4°C), the cAMP concentrations in the supernatants were quantified via ELISA according to the manufacturer’s protocols (Ruixin Biotechnology, China). The absorbance at 450 nm was measured with a microplate reader, and the biofilm cAMP content was normalized to the sample biomass using a standard curve-derived calibration.

To investigate the effects of cAMP signaling and the *liaS* gene on early fungal adhesion, dual-species biofilms of *C. albicans* and liaS^–^ were established through gradient supplementation of exogenous dibutyryl-cAMP (db-cAMP, Macklin Biochemical Co., Ltd., Shanghai, China) for functional complementation (Bernardoni et al., 2024). The fungal early adhesion model was developed under anaerobic conditions (37°C, 80 rpm, 8 h), with adherent fungal biomass quantified via CFU counting (Nabert-Georgi et al., 2018). Fungal morphology was assessed using scanning electron microscopy (SEM). Circular sterile glass slides (radius = 0.6 cm) served as carries for the biofilm, which was cultured in a 24-well plate. After rinsing thrice with PBS, the biofilm was fixed with a 2.5% glutaraldehyde solution (J&K Scientific, Co., Ltd., China). It was then dehydrated in a graded ethanol series, dried, and sputter-coated with gold-palladium. SEM images were randomly captured at 2,000× magnification using an EM8000 microscope (KYKY, China).

### Rat model of dental caries

Thirty SPF-grade male Sprague–Dawley rats (3 weeks old, 50 ± 5 g) were randomly allocated to five groups (*n* = 6): *WT-C. a*, *WT-S. m* 593, liaS^–^, *WT-C. albicans* + *WT-S. m* 593, and liaS^–^ + *WT-C. a* infection. Three days before infection, the rats received sterile water containing 0.1% (w/v) ampicillin, 0.1% (w/v) streptomycin, and 0.1% (w/w) carbenicillin to deplete the oral microbiota. Starting on day 5, the oral cavities were inoculated with 10^8^ CFU/ml *S. mutans* or 10^6^ CFU/ml *C. albicans* suspensions by swabbing for three consecutive days. Throughout the 21-days experiment, the animals were maintained on 5% sucrose water to promote cariogenesis, and their body weights were monitored. After euthanasia by cervical dislocation, the mandibles were aseptically dissected and homogenized in PBS using an ultrasonic processor. Serial dilutions were plated on mitis salivarius-bacitracin agar (MSBA, Difco, Biosciences) supplemented with 200 U/ml bacitracin and 1% potassium tellurite, followed by 48 h of anaerobic incubation at 37°C for *S. mutans* quantification. Caries progression was evaluated using the Keyes scoring system (Jafri et al., 2020; Chen et al., 2024). The rat teeth were stained with 0.4% ammonium purpurate for 12 h and graded under a stereomicroscope (ZEISS, Germany) for enamel (E), superficial dentin (Ds), and deep dentin (Dd) lesions.

The animal experiments were performed in strict accordance with the guidelines of the Animal Welfare Act by the ethics committee of Fujian Medical University and meet the ethical requirements (license number IACUC FJMU 2024-0378). This experiment met humane standards and minimizes the subject’s pain.

### Statistical analysis

All the experiments were conducted in triplicate individually. Significant effects of the variables were determined by one-way ANOVA, followed by Tukey’s multiple comparison tests. Differences were considered statistically significant if *P* < 0.05. Statistical analysis was performed using SPSS 16.0 software (SPSS Inc., Chicago, IL, USA).

## Results

### *LiaS* is a regulatory factor for the formation of biofilms

**FIGURE 1:**
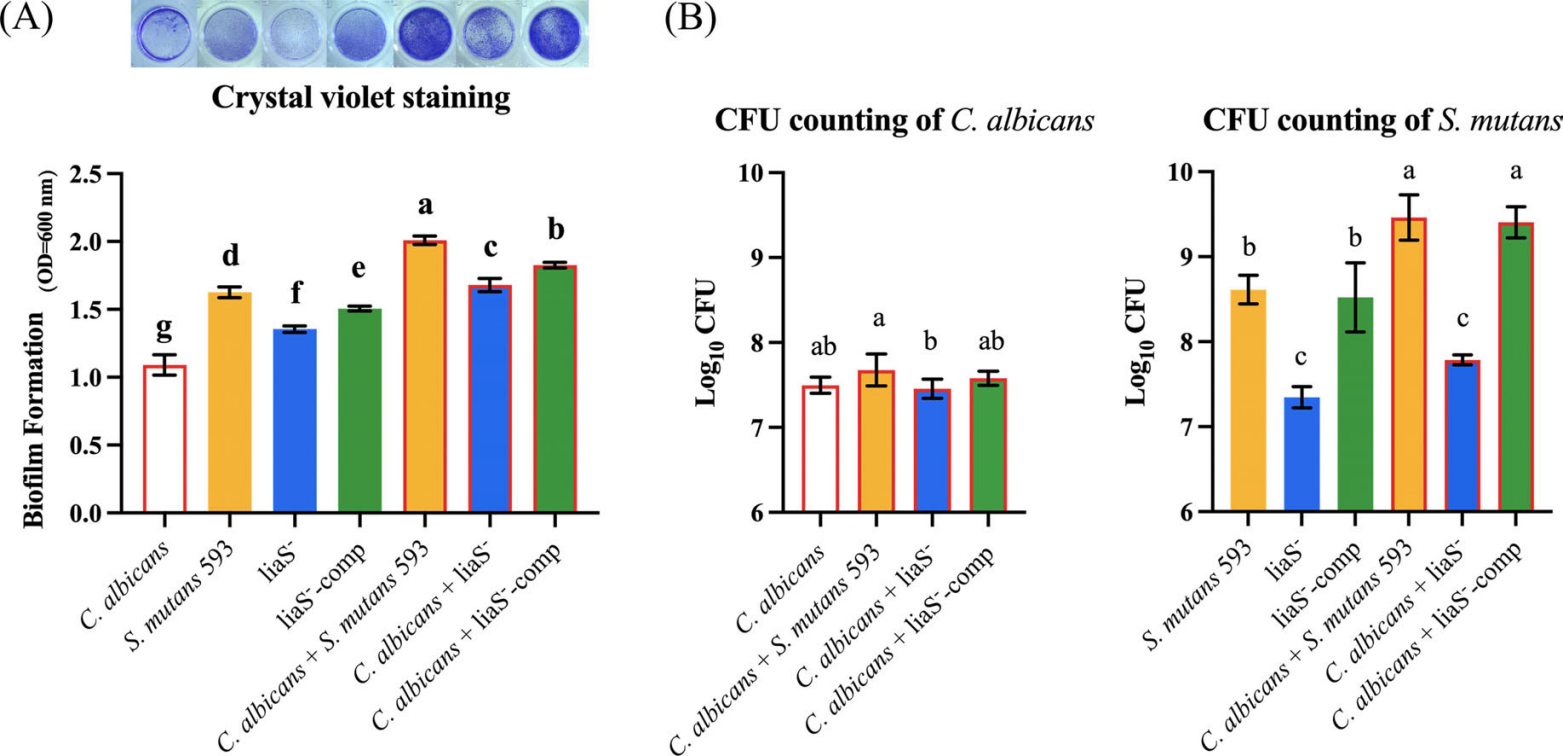
Single and dual-species of *S. mutans* and *C. albicans* biofilm biomass determined by CV assay and CFU count. **(A)** Quantitative analysis of biofilm biomass by CV assay with inserted panel of representative single and dual-species biofilm stained with CV. The result showed obvious inhibit effect of *liaS* gene on single and dual-species biofilms; **(B)** CFU counting of *S. mutans* and *C. albicans* in biofilms after 24 h. Data were presented in mean ± standard deviation and values with dissimilar letters were significantly different from each other; *P* < *0.05*.

Biofilm quantification revealed that the *liaS* gene promotes biofilm formation in both single and dual species films (Figure 1A). Compared with those of the *WT* and liaS^–^-comp strains, the percentages of liaS^–^ single-species decreased by 16.74% and 10.04%, respectively (*P* < 0.05). In the dual-species system, the combination of *liaS*-deficient samples showed significant reductions of 16.42% and 8.04% compared to the combination of *WT*-S. m and liaS^–^-comp, respectively (*P* < 0.05). Biofilm formation was restored by supplementation with the *liaS* gene. There was no significant difference in fungal biomass between the single and dual-species biofilms of *C. albicans*. The bacterial biomasses in the single and dual-species biofilms of the *liaS*-deficient strain were 7.35 and 7.79 log_10_ CFU/ml, respectively, which was significantly lower than those of the *WT* and liaS^–^-comp strains (*P* < 0.05; Figure 1B).

### *LiaS* governs the acid stress response and metabolic regulation

**FIGURE 2:**
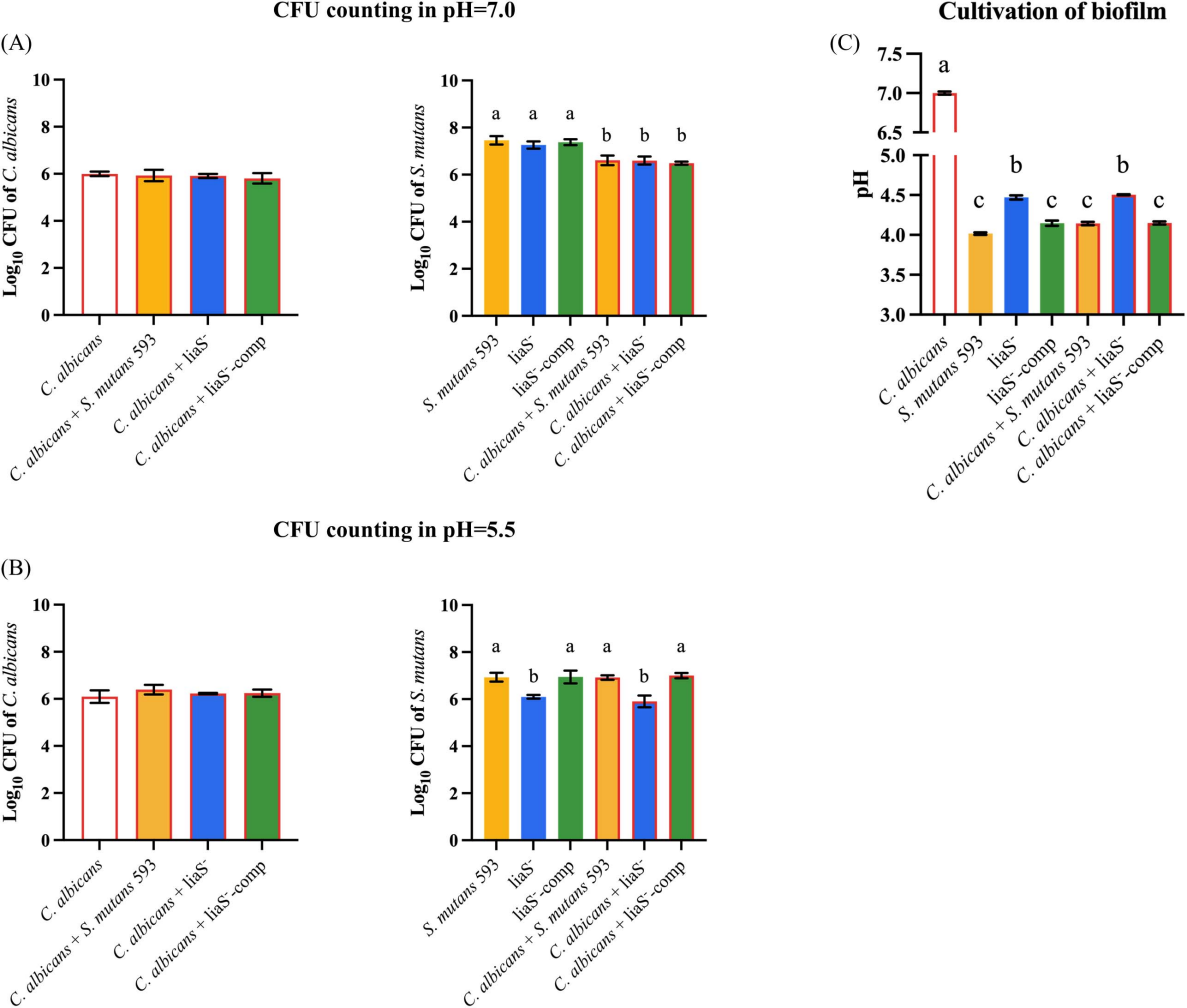
The effect of *liaS* on the growth and acid production function of single and dual-species under acid stress. **(A,B)** CFU counting of *C. albicans* and *S. mutans* in planktonic microorganisms in pH 7.0 and 5.5; **(C)** The pH value of supernatant culture medium. Data were presented in mean ± standard deviation and values with dissimilar letters were significantly different from each other; *P* < 0.05.

Planktonic cultures demonstrated that *C. albicans* maintained stable biomass under both neutral (pH 7.0) and acidic (pH 5.5) conditions (Figures 2A, B). In contrast, *S. mutans* exhibited pH-specific growth patterns: while the *WT* and liaS^–^ strains showed comparable proliferation at neutral pH (6.10 ± 0.08 vs. 5.91 ± 0.25 log_10_ CFU/mL; *P* > 0.05), acid stress (pH 5.5) significantly attenuated liaS^–^ growth in both mono- and dual-species cultures compared with the *WT* (*P* < 0.05). Additionally, in the 24-h planktonic culture system of the *liaS* gene knockout strain, the biomass was significantly lower than that of the *WT*-*S. m* and liaS^–^-comp strains (*P* < 0.05). Metabolic profiling revealed impaired acidogenesis in liaS^–^ cultures: after 24 h of cultivation, liaS^–^ mono- and dual-species supernatants presented an attenuated pH reduction (10.07% and 8.00% less acidification, respectively) compared with the *WT* strain (Figure 2C). This metabolic deficit was rescued in the complemented strain (liaS^–^-comp), and there was no significant difference in the pH value of the supernatant culture medium between single-species and dual-species biofilms.

### *LiaS* reduces EPS production and colony aggregation to inhibit biofilm formation

**FIGURE 3:**
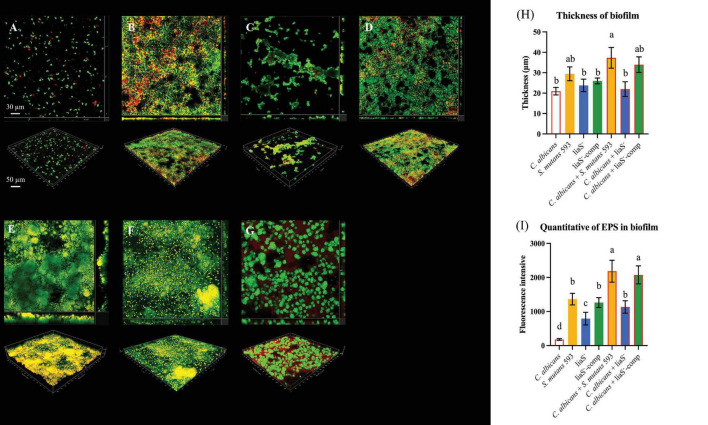
Observation of single-species and dual-species biofilms by CLSM. **(A–D)** 3D structure of single-species biofilm: *C. albicans*, *S. mutans* 593, liaS^–^, liaS^–^-camp; **(E–G)** 3D structure of dual-species biofilm: *C. albicans* and *S. mutans* 593, *C. albicans* and liaS^–^, *C. albicans* and liaS^–^-comp; **(H)** The mean thickness of both single-species and dual-species biofilms formed by *C. albicans* and *S. mutans*, which were cultured for a duration of 24 h, was precisely quantified utilizing IMARIS V9.0 software; **(I)** Quantitative analysis of EPS in single and dual-species biofilms. Data were presented in mean ± standard deviation and values with dissimilar letters were significantly different from each other; *P* < 0.05. (Bar = 50 μm).

The 3D reconstruction of biofilms and EPS distribution revealed significant structural alterations in liaS^–^. The single-species liaS^–^ biofilms presented a 19.3% reduction in the average thickness (23.79 μm), with complementation restoring the thickness by 11.9% (25.97 μm) compared with that of *WT*-*S. m* (Figures 3B–D). In dual-species cultures, liaS^–^ biofilms were 41.1% thinner (21.96 μm) than *WT* biofilms were (*P* < 0.05), whereas liaS^–^-comp biofilms presented minimal disparity (Figures 3E–G). Confocal imaging revealed that *C. albicans* and *S. mutans* (SYTO 9-green) formed dense aggregates with abundant EPS (red fluorescence) along fungal surfaces, whereas liaS^–^ displayed sparse bacterial clustering with reduced aggregation density and less EPS production (Figures 3C, F). Quantitative analysis of red fluorescence within the biofilm revealed that the *liaS* gene promotes EPS production (Figure 3I). Notably, the combination of liaS^–^ and *C. albicans* inhibited fungal hyphae dominance, suggesting an altered fungal-bacterial interplay. The complementation strain restored spatial organization and EPS synthesis patterns.

### LiaS^–^ downregulation of *sodA* leads to an increase in the intracellular ROS content

**FIGURE 4:**
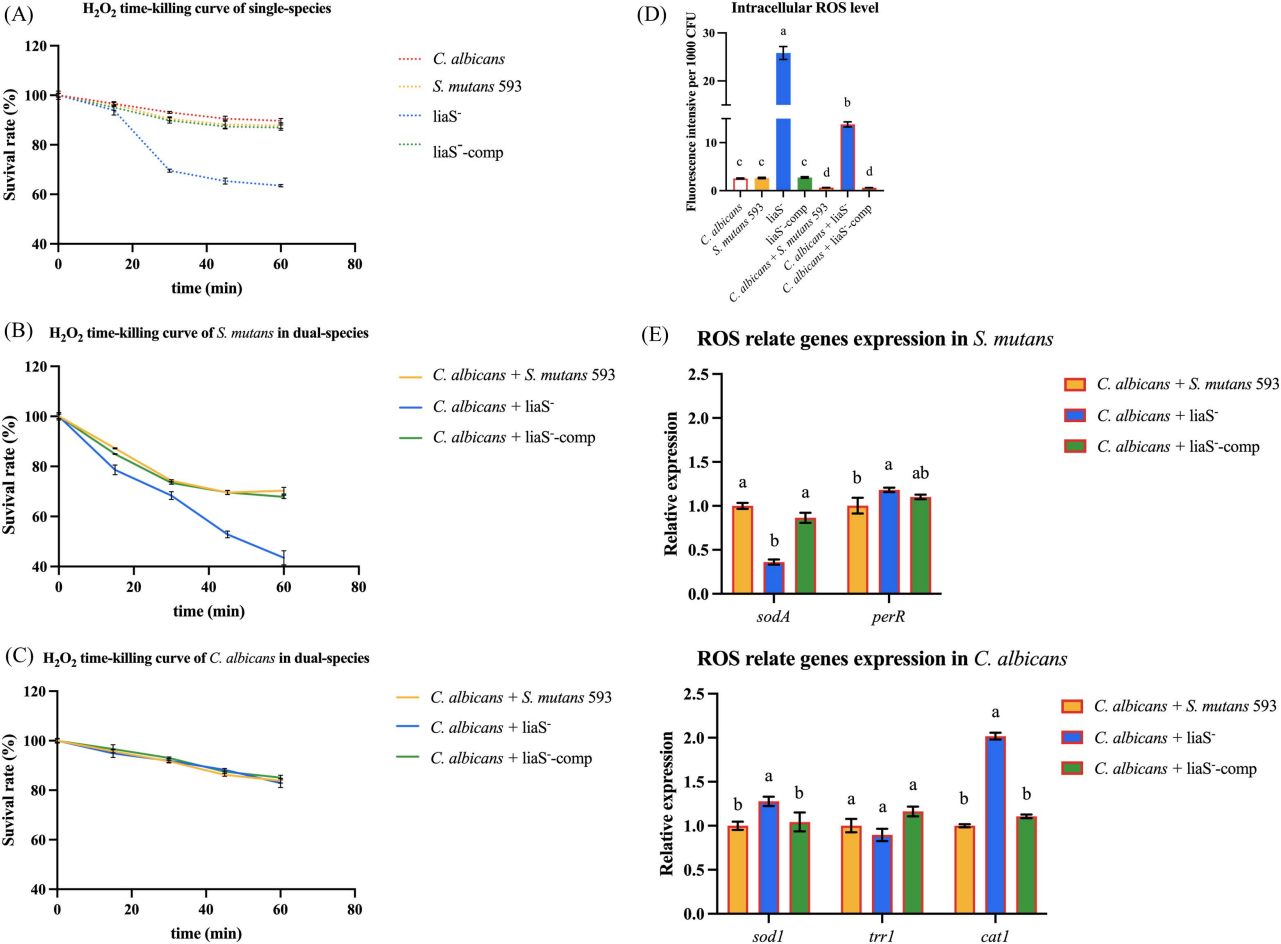
H_2_O_2_ time-killing curve and intracellular ROS content of biofilms. **(A–C)** The survival rates of *C. albicans* and *S. mutans* in mature biofilms of single and dual-species exposed to H_2_O_2_ for 60 min at different time are presented in percentage (%); **(D)** Intracellular ROS content of biofilm; **(E)** The expression level of antioxidant stress relate genes in *S. mutans* and *C. albicans*. Data were presented in mean ± standard deviation and values with dissimilar letters were significantly different from each other; *P* < 0.05.

Biofilms exposed to 2× MIC H_2_O_2_ presented strain-dependent survival kinetics (0–60 min). After being exposed to H_2_O_2_ for 60 min, *C. albicans* demonstrated superior resistance in both single-species (10.32% reduction) and dual-species (16.17%–17.09% reduction) strains (Figures 4A, B). Compared with those of the *WT* and liaS^–^-comp (*P* < 0.05) (Figure 4A), with exacerbation under dual-species conditions (21.39%–56.52% reduction) (Figure 4C). The liaS^–^ culture accumulated significantly higher intracellular ROS than *WT*-*S. m* and liaS^–^-comp both in single and dual-species biofilms (*P* < 0.05). Cocultivation with *C. albicans* reduced the level of endogenous ROS by 46.54%, in contrast to the results in single-species cultures (Figure 4D). *liaS* displayed cross-kingdom transcriptional regulation in dual-species biofilm, accompanied by 63.85% downregulation of *sodA* (*P* < 0.05) and an 18.17% upregulation of *perR* (*P* < 0.05) in *S. mutans*. In *C. albicans*, 27.72% of the *sod1* genes were upregulated (*P* < 0.05), 10.38% of the *trr1* genes were downregulated (*P* > 0.05), and 101.83% of the *cat1* genes were upregulated in *C. albicans*. This difference in gene expression regulation was reversed in liaS^–^-comp (Figure 4E).

### cAMP mediates early fungal adhesion in cross-kingdom biofilms

**FIGURE 5:**
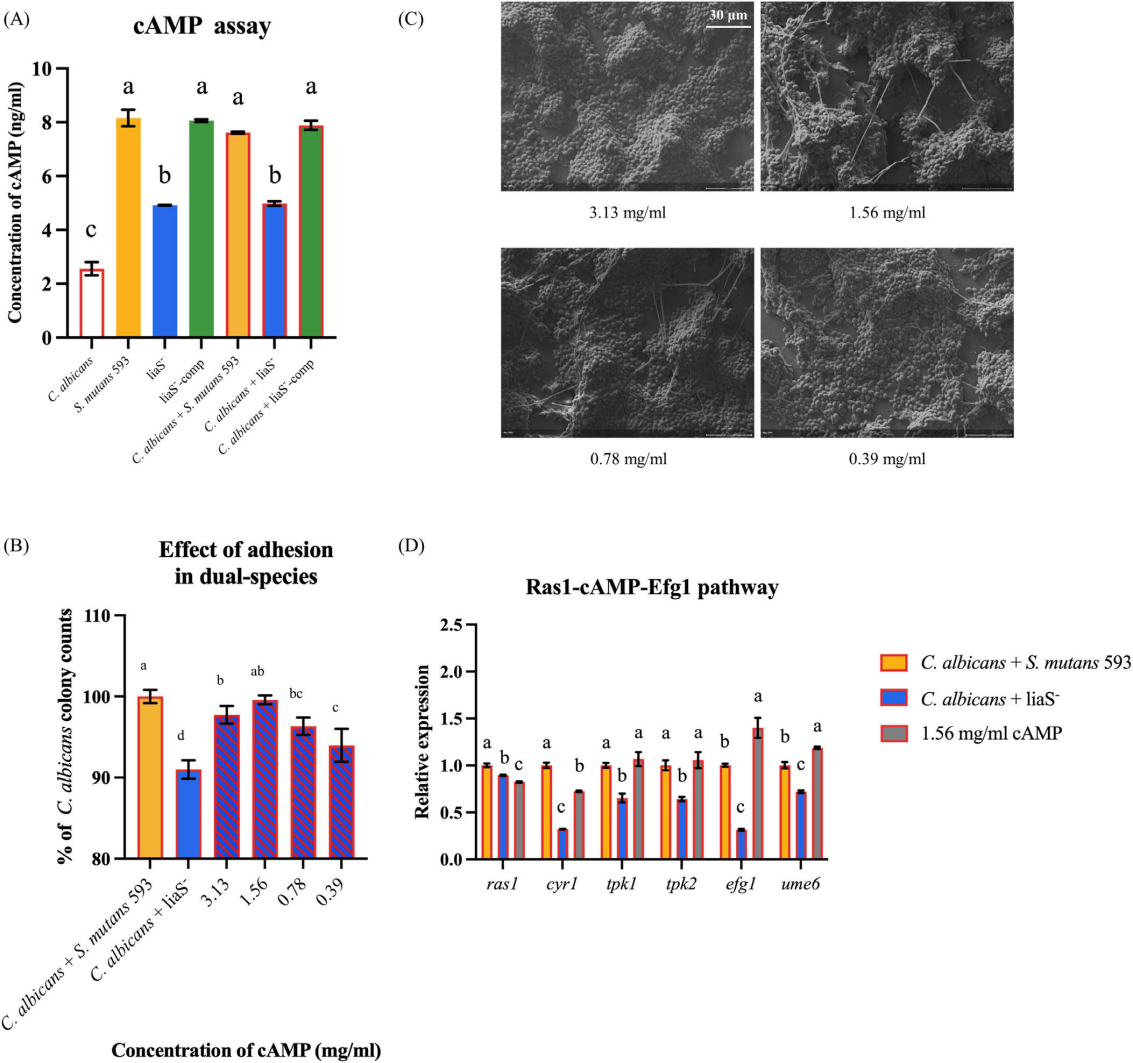
The content of cAMP in biofilm and the rescue experiment of db-cAMP on single-species and dual-species biofilms. **(A)** The quantitative of cAMP in biofilm; **(B,C)** The fungal early adhesion and hyphal formation in dual-species biofilm with *C. albicans* and liaS^–^; **(D)** Transcriptional and functional analysis of the Ras1-cAMP-Efg1 signaling axis in dual-species biofilms. Data were presented in mean ± standard deviation and values with dissimilar letters were significantly different from each other; *P* < 0.05. (Bar = 30 μm).

Quantitative cAMP analysis revealed that *liaS* had a regulatory effect on metabolites in the biofilms (Figure 5A). In *S. mutans* 593 single-species culture, the cAMP content (8.16 ng/ml) was 1.66-fold greater than in liaS^–^ (4.92 ng/ml) (*P* < 0.05). Compared with the single-species mixture, the liaS^–^ dual-species mixture did not present with significant differences in cAMP contents compared to the single species culture (*P* < 0.05). Early fungal adhesion in liaS^–^ and *C. albicans* biofilms depends on the dosage of db-cAMP. Compared with the combination of *S. mutans* 593 and *C. albicans*, exogenous supplementation with 1.56 mg/ml db-cAMP restored early adhesion, but the difference was not significant (Figure 5B). Notably, 1.56 mg/ml db-cAMP reversed these perturbations, significantly rescuing the early contribution of fungi in the liaS^–^ and *C. albicans* combination to the formation of complex biofilm structures (Figure 5C).

Regulatory profiling of the Ras1-cAMP-Efg1 signaling pathway in the *liaS*-deficient strain in dual-species biofilms revealed significant alterations in the expression of key genes governing the Ras1-cAMP-Efg1 cascade in *C. albicans* (Figure 5D). Specifically, the upstream regulator *ras1* was downregulated by 10.4% compared with that of the *WT* (*P* < 0.05). The adenylate cyclase-encoding gene *cry1* presented a 67.7% reduction in expression. The cAMP-dependent protein kinase (PKA) catalytic subunit genes *tpk1* and *tpk2* were downregulated by 34.6% and 35.8%, respectively. The core hyphal development transcription factor *efg1* and the biofilm regulatory gene *ume6* presented 68.5% and 27.9% reductions in transcript levels, respectively. Exogenous supplementation with 1.56 mg/ml db-cAMP restored the expression of this pathway in the *liaS*-deficient dual-species system, with statistically significant upregulation observed across pathway components (*P* < 0.05).

### *LiaS* gene participates in TCS expression collaboration

**FIGURE 6:**
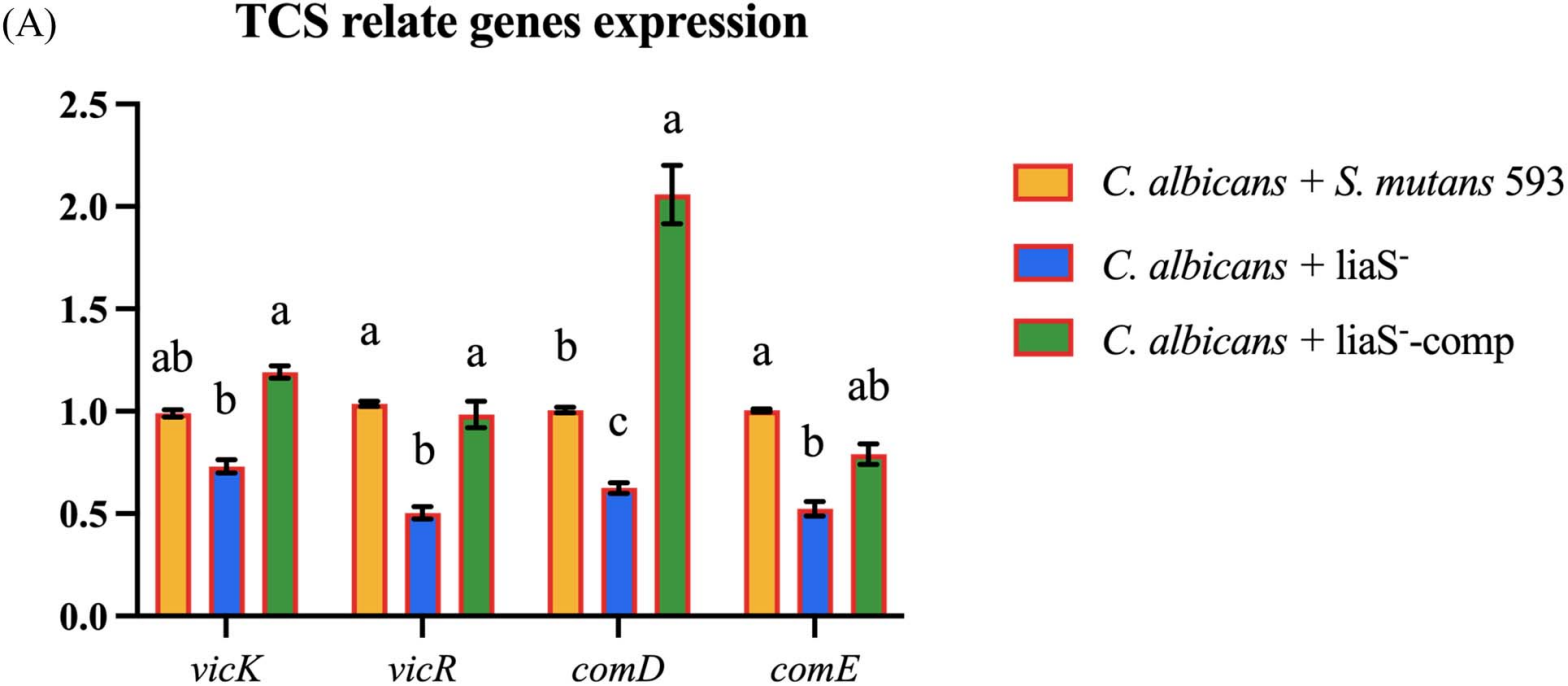
**(A)** The gene expression related to the TCS in cross-kingdom biofilm. Data were presented in mean ± standard deviation and values with dissimilar letters were significantly different from each other; *P* < 0.05.

Compared with the *WT*-*S. m*, liaS^–^ exhibited significant transcriptional downregulation of virulence regulators (*vicR*: −26.93%; *vicK*: −49.64%) and (*comD*: −37.48%; *comE*: −47.66%). All expression deficits showed partial or full recovery after *liaS* complementation (Figure 6A).

### *LiaS* promotes dental caries in rats

**FIGURE 7:**
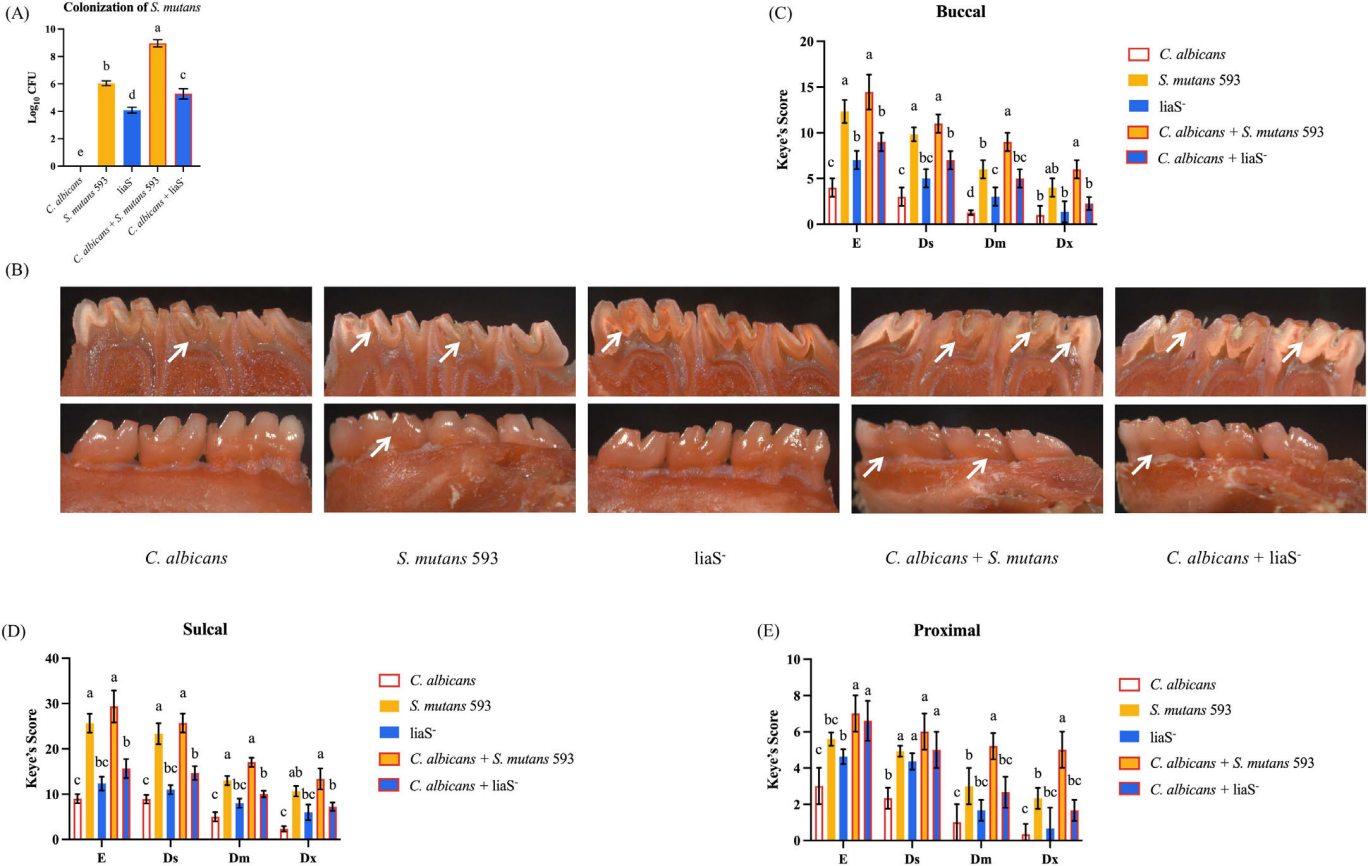
*LiaS* gene regulates the development of dental caries by *S. mutans* and *C. albicans*. **(A)** Colonization of *S. mutans* in rat oral plaque (*n* = 6); **(B)** Schematic diagram of dental caries on the lingual and buccal sides of rat mandibular molars, with the location of caries indicated by white arrows; **(C–E)** Keyes caries assessment classified the buccal, sulcal, and proximal of rat mandibular molars according to the severity of caries: E, Ds, Dm, and Dx. Data were presented in mean ± standard deviation and values with dissimilar letters were significantly different from each other; *P* < 0.05.

Quantitative analysis using the Keyes scoring system in Sprague–Dawley rats demonstrated that *liaS* is a critical regulator of *S. mutans*-mediated cariogenesis. The single-species and dual-species colonization of *S. mutans* loads were 6.045, 4.085, 8.965, and 5.278 log_10_ CFU/ml, respectively (Figure 7A). Keyes scores, which evaluate lesion depth and spread degree, revealed stark contrasts in caries severity (Figure 7B). The *WT*-*S. m* 593 infections induced dominant Dx lesions, with dual-species infections resulting in accelerated caries progression in the buccal surfaces, fissures, and proximal tooth regions (Figures 7C–E). In contrast, liaS^–^ infections significantly reduced the incidence of Dx lesions (*P* < 0.05) across both single-species and dual-species infections. Notably, the liaS^–^ and *C. albicans* combined infection displayed caries patterns that were phenotypically aligned with those of *C. albicans* single infection, which demonstrated minimal cariogenic activity.

## Discussion

As a keystone pathogen in oral diseases, *S. mutans* regulates intricate polymicrobial interactions with *Staphylococcus aureus*, *Porphyromonas gingivalis*, *Streptococcus sanguinis*, *Fusobacterium nucleatum*, and *C. albicans*, collectively driving the pathogenesis of dental caries, periodontal disorders, and peri-implant infections through cross-species metabolic synergy and spatial co-organization (Wang and Ren, 2017; Nabert-Georgi et al., 2018; Elzayat et al., 2023; Bernardoni et al., 2024). The cariogenic arsenal of *S. mutans* comprises four cardinal mechanisms: (a) EPS-mediated biofilm nucleation and architectural stabilization, (b) bacteriocin-driven ecological competitiveness, (c) acidogenic/aciduric carbohydrate metabolism, and (d) stress-responsive phenotypic plasticity. TCS systems serve as master regulators of these pathogenic cascades, integrating environmental cues with virulence determinant expression. While mono-species experimental models have established the LiaSR TCS system as a pivotal controller of *S. mutans* biofilm maturation and stress adaptation, the inherent ecological complexity of clinical oral environments, particularly those colonized by opportunistic fungal species such as *C. albicans*, demands transformative re-examination of TCS-related operational dynamics in multispecies consortia. This biological imperative underscores the critical need to elucidate how the LiaSR governs virulence adaptation within polymicrobial structured biofilm regulatory networks.

**FIGURE 8:**
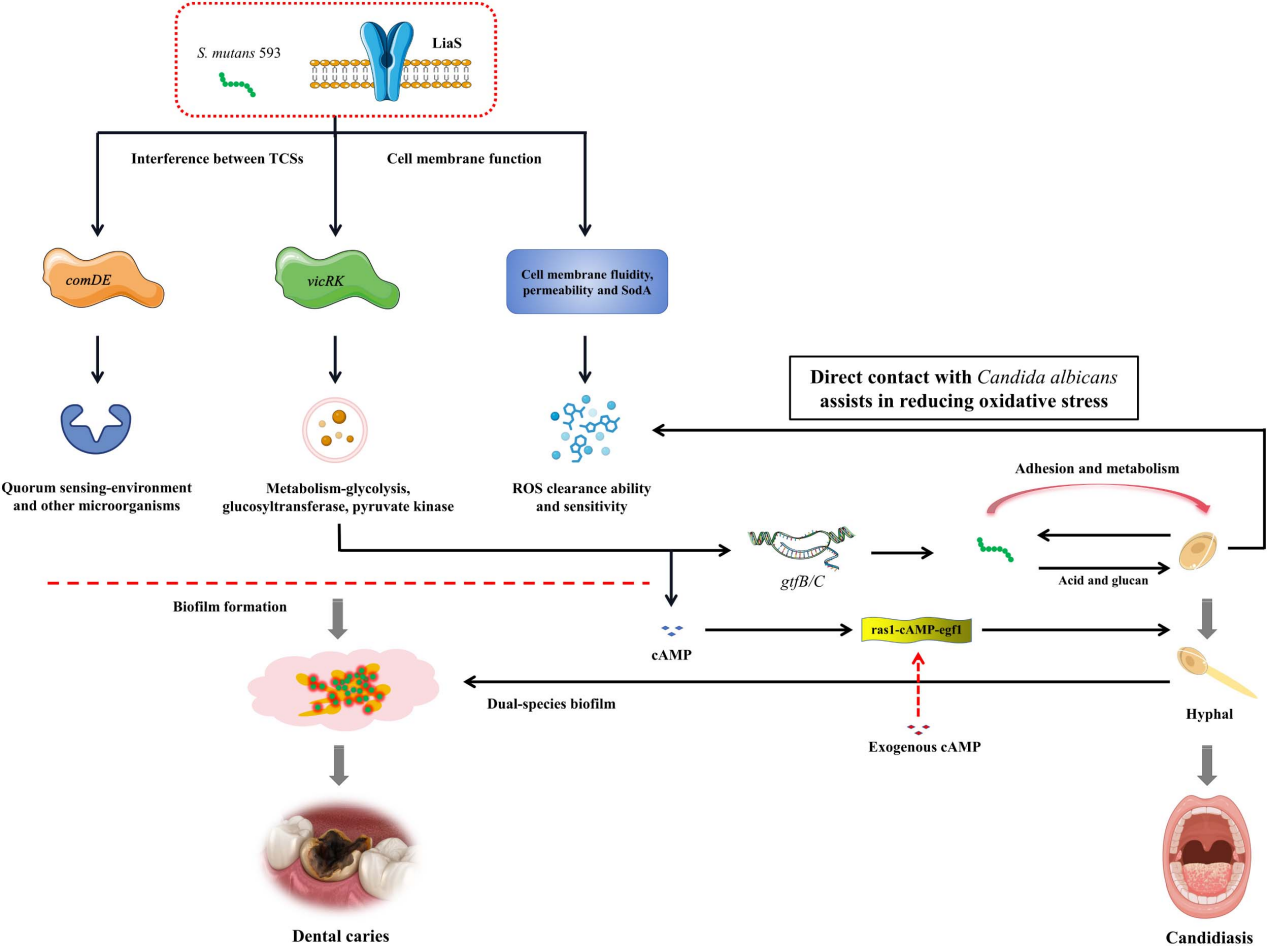
The mechanism of *liaS* regulating the cariogenic of *S. mutans* and its cross-kingdom interaction with *C. albicans*.

Our study systematically compared *WT*-*S. m* 593 and liaS^–^ strains in dual-species biofilm models with *C. albicans*. The results revealed that *liaS* is a master regulator that governs both architectural maturation and dynamic structural remodeling of cross-kingdom biofilms. Mechanistically, transcriptomic profiling revealed that *liaS* deletion disrupts the reciprocal modulation between *vicRK* and *comDE* TCS (Figure 8), thus impairing acid stress adaptation, metabolic acidogenesis, and EPS-dependent structural synergy. Additionally, *LiaS* further coordinates oxidative stress homeostasis by regulating redox-related genes in *S. mutans* and cross-kingdom interactions with those in *C. albicans*, whereas cAMP-mediated signaling underpins its role in early fungal adhesion. *In vivo*, *liaS* deletion attenuated the cariogenicity of *S. mutans*, even in dual-species infections, thus phenocopying the non-pathogenic biofilm behavior of *C. albicans* alone. These findings collectively position *LiaS* not only as a central regulator of *S. mutans* virulence but also as an orchestrator of both intrinsic stress responses and interkingdom collaboration to drive polymicrobial biofilm pathogenicity and dental caries progression.

The molecular interplay between *S. mutans*-derived water-insoluble glucans and fungal mannoproteins establishes a critical pathogenic nexus, thus facilitating the assembly of cross-kingdom biofilm composites through stereospecific lectin-carbohydrate interactions (Ren et al., 2022). Our quantitative assays demonstrated that this synergy elevates the biomass of *WT*-*S. m* in dual-species cocultures compared with liaS^–^ and liaS^–^-comp while enhancing plaque mechanical stability in murine caries models (Li et al., 2008). These findings indicate that *liaS* serves as the core of this process. Mechanistically, *vicRK* regulates EPS biosynthesis via direct binding to the *gtfB* promoter region, concurrently modulating virulence determinants, including BrpA-mediated biofilm structural remodeling and *comDE*-regulated interspecies signaling molecules (Abranches et al., 2018; Ren et al., 2022). Notably, while previous studies delineated *vicRK*-*comDE* crosstalk in single-species contexts, our work revealed unprecedented *liaS*-dependent regulation of TCS network dynamics in polymicrobial environments. CLSM coupled with 3D biofilm reconstruction revealed that *liaS* deletion reduces bacterial-fungal co-adhesion and EPS matrix density (Figure 3). These structural perturbations are correlated with dysregulated expression of both *vicRK* and *comDE* operons in liaS^–^, as confirmed by RT-PCR (Figure 6). This signaling imbalance can suppress glucosyltransferase gene expression and fungal β-glucan adhesin synthesis, thus creating a feedback loop that destabilizes the biofilm matrix (Abranches et al., 2018).

The pathogenesis of dental caries stems from the microbial utilization of environmental sugars as substrates, wherein the phosphoenolpyruvate-dependent phosphotransferase system (PEP-PTS) catalyzes the transport and phosphorylation of monosaccharides, disaccharides, amino sugars, polyols, and other sugar derivatives, ultimately generating substantial amounts of organic acids (Lu et al., 2023). In *liaS*-deficient cultures, the levels of acidic metabolites were significantly reduced (Figure 2C). These findings corroborated our previous findings on *liaS*-mediated aciduricity regulation, confirming its dual role as a master regulator in stress adaptation (Huang et al., 2024). The acidic microenvironment acts as a mediator, amplifying the synergistic metabolic interplay within dual-species biofilms of *S. mutans* and *C. albicans*, thereby intensifying cariogenic pathology (Li et al., 2018). Compared with single-species infections, dual-species biofilms present increased hyphal density, elevated EPS production, and the formation of densely packed 3D structures, thereby collectively fostering the development of an acidic niche that accelerates demineralization of hard dental tissues (Krzyściak et al., 2014; Pallavi et al., 2024).

The *liaSR* exerts central regulatory control over this cross-kingdom interaction. Previous studies have demonstrated that *liaS*-deletion elevates the lethal pH threshold, reduces H^+^-ATPase activity, and severely impairs the adhesive capacity of *S. mutans* biofilms (Feldman et al., 2016; Huang et al., 2024). Our findings extend these observations to polymicrobial biofilm microenvironments: compared with *WT*-*S. m* 593 cocultures with *C. albicans*, the liaS^–^ coculture system exhibited attenuated EPS synthesis and hyphal morphogenesis *in vitro* (data not shown), significantly reduced Keyes scoring indices and alleviated lesion severity in rat models.

The oral biofilm formed by *S. mutans* establishes a protective barrier for cariogenic microbial communities, shielding them against antimicrobial agents and environmental stressors. Intriguingly, *C. albicans* exhibited adaptive compensation in coculture systems: the upregulation of *sod1* (superoxide dismutase) and *cat1* (catalase) expression effectively neutralized biofilm-associated ROS. These findings suggest that fungi may function as biological shields to protect *S. mutans* from oxidative damage through a mechanism dependent on direct cell-to-cell contact (Gregoire et al., 2011; Čížová et al., 2019; Katrak et al., 2023; Yan et al., 2023). Bidirectional regulatory nodes govern these interactions: the *liaSR* system in *S. mutans* not only modulates its TCS network to regulate autonomous EPS synthesis and competitive fitness but also promotes hyphal transition in *C. albicans*. Conversely, *chk1*-deficient mutants of *C. albicans* exhibit analogous suppression of cross-kingdom interactions, thus suggesting the existence of biosignaling communication mechanisms between these microorganisms (Coates et al., 2020; Liu et al., 2022). Therefore, therapeutic strategies targeting cross-kingdom cariogenic synergism, particularly through the inhibition of hyphal morphogenesis and EPS production, represent a promising direction for exploration.

The mechanistic analysis in this study revealed that *liaS* orchestrated fungal-bacterial synergism through the modulation of TCS expression levels. Specifically, *liaS* mediated the perturbation of TCS homeostasis in *S. mutans* by modulating the *vicRK* and *comDE* signaling axes in biofilms, as well as cross-kingdom interference with fungi. Among these regulatory mechanisms, the *vicRK* operon activates downstream genes essential for biofilm maturation and acid tolerance, specifically binding to the promoter regions of *gtfB* and *gtfC*, driving EPS synthesis and mediating genetic interactions in cross-kingdom biofilms (Ellepola et al., 2019). CSP activates the ComD transmembrane receptor (encoded by the *comD* gene), triggering the phosphorylation cascade of the QS core genes *comE* and *sigX*. This regulatory axis not only increases the acidogenicity of *S. mutans* but also inhibits competing species through the production of mutacin IV, a bacteriocin that targets gram-positive species (Zu et al., 2019). This cross-kingdom phenotype change included downregulating the gene expression of ras1-cAMP-efg1 in the hyphal transition pathway, reducing extracellular glucan-binding protein production, and diminishing biofilm cariogenicity by impairing synergistic sucrose-driven EPS and matrix co-deposition. Crucially, the complementarity of db-cAMP restored the early adhesion ability of fungal cells (Figure 5), thus confirming the centrality of the *liaS* gene in mediating cross-kingdom cariogenic partnerships. cAMP, which serves as a vital second messenger within cells, plays a pivotal role in the formation of biofilms and the cariogenic process in *S. mutans*. It exerts its influence by activating or inhibiting GTFs, thereby modulating EPS synthesis and adhesion to other microorganisms (Konno et al., 2018; Lin et al., 2022b; Rojas et al., 2024).

Consequently, multidimensional disruption of these cooperative mechanisms may yield novel strategies for caries control and polymicrobial infection management. Critical knowledge gaps persist, particularly with respect to deciphering the molecular architecture of TCS crosstalk. These findings should be interpreted in light of certain limitations, including the reliance on single-strain models of *C. albicans* SC5314 that may not fully represent clinical heterogeneity, as well as the simplified dual-species biofilm system, which excludes broader ecological interactions within native oral microbiomes. Future investigations employing phosphoproteomics and structural analyses are imperative to elucidating how *liaSR* coordinates with the *vicRK* and *comDE* systems through phosphorylation cascades and how the cAMP content mediates fungal-bacterial signal integration. Additionally, a deeper exploration of strain-specific behaviors, host-microbe interplay, and temporal dynamics of biofilm maturation will refine translational relevance. Unraveling these mechanisms will enable precise targeting of evolutionarily conserved nodes within microbial interaction networks.

## Conclusion

*LiaS* deficiency mediates cariogenic pathogenesis through two regulatory mechanisms: (1) downregulating *vicRK* and *comDE* to reduce EPS biosynthesis and inhibit biofilm development and (2) modulating cross-kingdom interactions with *C. albicans* to increase the structural complexity of dual-species biofilms and increase virulence. Our findings established *liaS* as a pivotal genetic determinant in the pathogenesis of caries in dual-species infections. Mechanistically, liaS^–^ not only impairs the ability of *S. mutans* to scavenge intercellular ROS but also exacerbates oxidative stress susceptibility in biofilms. These collective insights position *liaS* as a promising therapeutic target for disrupting the fungal-bacterial collaborative networks underlying the progression of dental caries.

## Data Availability

The data presented in the study are deposited in the Zenodo repository, accession number: 10.5281/zenodo.16108326.
