# Supplementary material for: LiaS gene from two-component system is essential for caries pathogenicity in dual-species biofilms of Streptococcus mutans and Candida albicans
Source: Front Microbiol. 2025 Jul 31;16:1612841. doi: 10.3389/fmicb.2025.1612841 (PMC12352329; doi:10.3389/fmicb.2025.1612841)
Supplement: Supplementary file 1 [file Table_1.docx]

**Supplementary Material**

Supplementary Table 1: Primers used for RT-PCR analysis of gene expression

| **Primers** | **Nucletide sequence (5’→3’)** |
| --- | --- |
| *16S RNA*-F | -AGCGTTGTCCGGATTTATTG- |
| *16S RNA*-R | -CTACGCATTTCACCGCTACA- |
| *18S RNA*-F | -CACGACGGAGTTTCACAAGA- |
| *18S RNA*-R | -CGATGGAAGTTTGAGGCAAT- |
| *sod1*-F | -GCAGTGCTAAGACTCCCGAATC- |
| *sod1*-R | -TTGCGGAAGTGTGGAGATTGGC- |
| *perR*-F | -CCTAGAGCATCTACGGGA- |
| *perR*-R | -CCGGACAAATCCCGTAAG- |
| *sodA*-F | -CTACTGATGGTAATGGTGTTGCTAA- |
| *sodA*-R | -CCAGCATGACCAGTAGTTTTAGAAT- |
| *trr1*-F | -TCATTGCTACTGGTGCCTCT- |
| *trr1*-R | -CAAGCTGAATCACCACCACC- |
| *cat1*-F | -GACTGCTTACATTCAAAC- |
| *cat1*-R | -AACTTACCAAATTTCTCA- |
| *vicK*-F | -CGTGAATTACTGGCGCGTGT- |
| *vicK*-R | -CAACTCAAACTCACGGTGAG- |
| *vicR*-F | -CACTTTACGCATTCGTTTTGCC- |
| *vicR*-R | -CGTTCTTCTTTTTCCTGTTCGGTC- |
| *comD*-F | -TTCCTGCAAACTCGATCATATAGG- |
| *comD*-R | -TGCCAGTTCTGACTTGTTTAGGC- |
| *comE*-F | -TTCCTCTGATTGACCATTCTTCTG- |
| *comE*-R | -GAGTTTATGCCCCTCACTTTTCAG- |
