# Supplementary material for: LiaS gene from two-component system is essential for caries pathogenicity in dual-species biofilms of Streptococcus mutans and Candida albicans
Source: Front Microbiol. 2025 Jul 31;16:1612841. doi: 10.3389/fmicb.2025.1612841 (PMC12352329; doi:10.3389/fmicb.2025.1612841)
Supplement: Supplementary file 2 [file Table_2.docx]

**Supplementary Material**

Supplementary Table 2: The MIC of H_2_O_2_ in time killing curves

| **Experimental group** | **Minimum inhibitory concentration (μg/mL)** |
| --- | --- |
| *C. albicans* | 117.2 |
| *S. mutans* 593 | 29.3 |
| liaS^-^ | 14.6 |
| liaS^-^-comp | 29.3 |
| *C. albicans + S. mutans* 593 | 234.4 |
| 1. *albicans +* liaS^-^ | 117.2 |
| *C. albicans +* liaS^-^-comp | 234.4 |
